# Therapeutic Maintenance Level of Methotrexate in Rheumatoid Arthritis: A RBSMR Study

**DOI:** 10.31138/mjr.33.2.224

**Published:** 2022-06-30

**Authors:** Lamia Oulkadi, Samira Rostom, Ihsane Hmamouchi, Imane El Binoune, Bouchra Amine, Redouane Abouqal, Lahsen Achemlal, Fadoua Allali, Imane El Bouchti, Abdellah El Maghraoui, Imad Ghozlani, Hasna Hassikou, Taoufik Harzy, Linda Ichchou, Ouafae Mkinsi, Radouane Niamane, Rachid Bahiri

**Affiliations:** 1 Department of Rheumatology A, El Ayachi Hospital, Ibn Sina University Hospital, Salé, Morocco,; 2 Laboratory of Biostatistical, Clinical and Epidemiological Research, Faculty of Medicine and Pharmacy, Mohammed V University, Rabat, Morocco,; 3 Department of Rheumatology, Provincial Hospital of Temara, Morocco,; 4 Department of Rheumatology, Military Hospital Mohammed V, Ibn Sina University Hospital, Rabat, Morocco,; 5 Department of Rheumatology B, El Ayachi Hospital, Ibn Sina University Hospital, Salé, Morocco,; 6 Department of Rheumatology, Arrazi University Hospital, Marrakech, Morocco,; 7 Private Medical Office, Rabat, Morocco,; 8 Department of Rheumatology, University Hospital of Agadir, Morocco,; 9 Department of Rheumatology, Military Hospital Moulay Ismail, Hassan II University Hospital, Meknès -Morocco,; 10 Department of Rheumatology, Hassan II University Hospital, Fès, Morocco,; 11 Department of Rheumatology, Mohammed VI University Hospital, Oujda, Morocco,; 12 Department of Rheumatology, Ibn Rochd University Hospital, Casablanca, Morocco,; 13 Department of Rheumatology, Military Hospital Avicenne, Mohammed VI University Hospital, Marrakech, Morocco

**Keywords:** methotrexate, rheumatoid arthritis, biotherapy registry, Morocco, rheumatology, therapeutic

## Abstract

**Objectives:**

The aim of this study was to determine the therapeutic maintenance level of methotrexate for rheumatoid arthritis patients enrolled in the Moroccan biotherapy registry and to identify predictive factors for discontinuing MTX treatment.

**Methods:**

A cross-sectional study was conducted using the baseline data of the Moroccan biotherapy registry for RBSMR (a multicentric study that aims to evaluate tolerance of biological therapy on patients affected with rheumatic diseases). Demographics and disease features were compared using descriptive statistics. Therapeutic maintenance levels were determined according to a Kaplan-Meier survival curve and a univariate Cox proportional hazards regression model was used to compare the strength of potential factors, followed by a multivariate Cox model to identify significant predictors of MTX discontinuation. Statistically significant results were considered for *p* values less than 0.05.

**Results:**

224 patients with rheumatoid arthritis were included in this study. The mean age of patients was 51.83±11.26 years with a majority of females (87.50%). The median duration of disease was 12 [1.66–41.02] years. The therapeutic maintenance level of MTX was 91.1% at 1 year, 87.1% at 2 years, and 68.3% at 5 years. The median of treatment duration was 2, 02 [0, 46–27,76] years. Causes of treatment interruption were side effects (66/88=75%), inefficiency (12/88=13.63%), and other reasons (10/88=11.36%). Predictive factors for stopping MTX were presence of rheumatoid factor (HR 2.24; 95% CI 1.14–5.15; *p*=0.02) and the access to education (HR 0.37; 95% CI 0.16–0.88; *p*=0.02).

**Conclusion:**

The therapeutic maintenance level of MTX in our study was satisfactory and comparable to other series, and influenced by many factors such as the occurrence of a side effect. It is necessary to sensitise medical practitioners on symptomatic prevention and management of side effects.

## INTRODUCTION

Rheumatoid arthritis (RA) is the most common chronic inflammatory rheumatic disease and affects 0.5–1% of the general population, with a predominance of women. RA can be the cause of a major functional handicap.^1^ The “Treat to Target” strategy has the ultimate goal of remission or at least low disease activity.^2^ Towards this end, a breakthrough has occurred in the therapeutic management of RA and in the dynamics of achieving clinical remission with the use of methotrexate (MTX).

Recommendations have been published^2,3^ for the optimal use of available therapeutics for the treatment of RA which include the administration of conventional synthetic disease-modifying antirheumatic drugs (csDMARDs) with methotrexate as the first line. MTX is an antifolate agent with anti-inflammatory and immunosuppressive properties at low doses.^4^ It is considered to be the drug of choice for treating RA because of its efficacy and safety both as a first-line monotherapy for treatment-naive patients,^5–7^ and in combination with other conventional or biological DMARDs for MTX-insufficient responders.^8–10^

MTX treatment should be maintained as long as possible in RA patients. MTX interruption poses a serious problem and can be associated with many factors. However, there is insufficient data on the therapeutic maintenance level of MTX and long-term tolerance in real-life clinical practice conditions. In addition, few studies have evaluated the factors associated with discontinuation of this therapy. The main objectives of this study were to determine the therapeutic maintenance level of MTX for patients included in the Moroccan biotherapy registry and to identify predictive factors for discontinuing MTX treatment.

## MATERIALS AND METHODS

### RBSMR Study

The RBSMR (Registre des Biothérapies de la Société Marocaine de Rhumatologie) is a registry of biological therapies in rheumatic diseases from the Moroccan Society of Rheumatology. It is a historical, prospective, and multicentre registry, which includes departments of rheumatology from 10 different university medical centres. The patients recruited in the registry were over the age of 18 years, diagnosed with RA or spondyloarthritis (SpA), and treated with biotherapy (initiation or ongoing biotherapy) in Moroccan university medical centres. The inclusion period was from May 2017 to January 2019 and the follow-up was 3 years. 440 patients were included in the study, in which 418 patients were validated (224 RA and 194 SpA). The primary objective of the RBSMR registry was to assess the tolerability of patients with RA or SpA to treatment by biotherapy in rheumatology. The secondary objectives were to identify the most common side effects in daily practice, to evaluate the effectiveness of biotherapies in rheumatology, and to evaluate the impact of biotherapies on the patients’ quality of life. The details of the data collected have been published previously.^11^

### Study Aims

We performed a cross-sectional, multicentre, analytical study using the RBSMR registry database which included 224 patients followed for RA. The objective of this study was to determine the therapeutic maintenance level of MTX at fixed durations, to analyse the causes of interruption of MTX, to identify predictive factors for stopping MTX, and to suggest some recommendations for increasing the therapeutic maintenance level of MTX.

### Pre-Treatment Characteristics

Collected characteristics were age, sex, level of education, and duration of disease. Clinical and para-clinical data relative to RA was assessed by the 28 joint count disease activity score (DAS28), presence of rheumatoid factor, erythrocyte sedimentation rate (ESR), and levels of anti-citrullinated protein antibodies (ACPA), C reactive protein.

### Data Relative to MTX Treatment

Information on the duration of treatment, the interruption of MTX, and the reason for this interruption was collected. Therapeutic maintenance level of MTX is defined as the percentage of patients continuing this treatment as a function of time.^12^ To calculate the therapeutic maintenance level, we consider the event of interest to be the permanent discontinuation of MTX at 1 year, 2 years, and 5 years, whatever the causation. Secondly, we analysed potential predictive factors for stopping MTX including clinical, biological, and demographic parameters: age, sex, educational level (illiterate, primary, secondary, post-secondary), social security (mandatory health insurance or medical assistance for people in economic decline), smoking habit, duration of RA, body mass index (BMI) >25 kg/m^2^, ESR >40, DAS 28-ESR, ACPA, rheumatoid factor (positive or negative), and corticosteroids and/or NSAIDs intake.

### Statistical Analysis

Statistical analysis was performed using SPSS software, version 13.0. Normally distributed parameters were presented as mean ± standard deviation (SD), and asymmetric parameters were expressed as median ± interquartile range (IQR, defined as 25–75th percentiles). Qualitative data were presented as frequencies (number and percentage). Therapeutic maintenance levels were determined according to a Kaplan-Meier survival curve. A univariate Cox proportional hazards regression model (Cox model) was used to estimate the potential factors of MTX discontinuation, followed by a multivariate Cox model to identify significant predictors of this discontinuation. Variables whose degree of significance in univariate analysis that was ≤ 0.50 were introduced into a multivariate Cox model. Statistical significance was considered for *p* values ≤ 0.05.

## RESULTS

### Patient Characteristics

A total of 224 RA patients were included in this study, which were predominantly female (n=196; 87.50%). The mean age of all subjects was 51.83±11.26 years. The median duration of disease was 12 [1.66–41.02] years. 99 (44.20%) patients were illiterate. The rheumatoid factor was positive in 191 patients (85.30%), and ACPA in 169 (75.50%). The majority of patients were active with a mean DAS28-ESR of 3.98±1.52. 199 (88.80%) patients received MTX and it was prescribed as a first-line treatment in 176 (78.60%) patients. A total of 193 (97.00%) patients were under corticosteroids (**Table 1**).

**Table 1.** Baseline characteristics of the study population.

**Parameters**	**Patients with RA (n=224)**	**Received MTX (n=199)**	**Remained on MTX (n=111)**	**Stopped MTX (n=88)**	***p* ^1^**
Age (years)^1^	51.83±11.26	51.15±11.24	49.58±12.40	53.14±9.78	0.02
Gender (female)^2^	196(87.50)	175(87.90)	97(87.40)	78(88.60)	0.78
Disease duration	12 [1.66–41.02]	13,92[1.67–41.02]	11,5[1.67–41.02]	13.03[3–40.02]	0.32
Smoking	4 (1,8)	3(1,5)	1(0,9)	2(2,3)	0.58
Educational level					
Illiterate^2^	99(44.20)	84(42.20)	44(39.60)	40(45.50)	0.40
Primary^2^	41(18.30)	38(19.10)	26(23.40)	12(13.60)	0.08
Secondary^2^	52(23.20)	48(24.10)	26(23.40)	22(25.00)	0.79
Post-Secondary^2^	22(9.80)	20(10.10)	8(7.20)	12(13.60)	0.13
BMI^1^ (kg/cm^2^)	27.55±5.08	27.50±4.90	27.24±4.63	27.83±5.23	0.42
MTX treatment^2^	199(88.80)	199(100)	111(55.80)	88(44.20)	-
MTX as a first line^2^	176(78.60)	176(88.40)	103(92.80)	73(83.00)	-
Corticosteroid treatment^2^	221(94.20)	193(97.00)	108(97.30)	85(96.60)	0.77
Rheumatoid factor^2^	191(85.30)	173(86.90)	99(89.20)	74(84.10)	0.42
ACPA^2^	169(75.50%)	166(78.40)	90(81.10%)	86(75.00)	0.27
DAS 28-ESR^2^	3.98±1.52	3.99±1,53	3.98±1.50	4.01±1.58	0.90
HAQ-DI^1^	1.22±0.74	1.16±0.73	1.25±0.74	1.04±0.70	0.09

ACPA, anti-citrullinated protein antibodies; BMI, body mass index; DAS, disease activity score; ESR, erythrocyte sedimentation rate; HAQ-DI, health assessment questionnaire disability index; MTX, methotrexate; RA, rheumatoid arthritis.

* Comparative analyses for patients remained on MTX and stopped MTX.

1 Mean and standard deviation;

2 Number and percentage.

### Therapeutic Maintenance Level of MTX

Of the 199 patients that received MTX, treatment had completely stopped in 88 patients (39.30%). The median of treatment duration was 2, 02 [0,46– 27,76] years.

**Figure 1.**
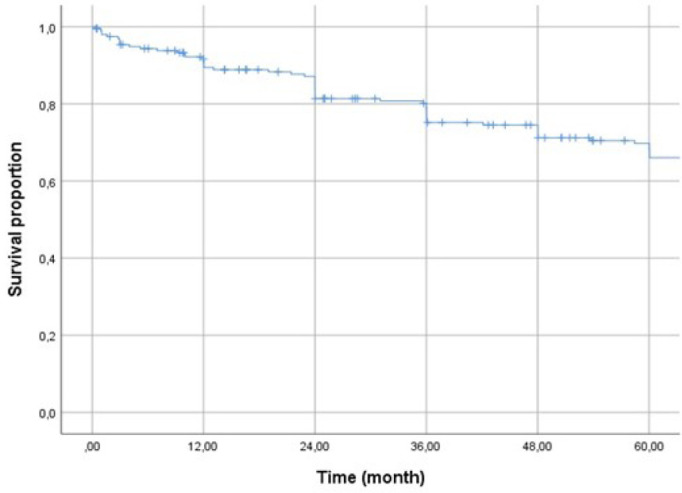
Cumulative probability for continuing methotrexate treatment.

The therapeutic maintenance level of MTX was 91.01% at 1 year, 87.10% at 2 years, and 68.30% at 5 years (**Figure 1**). The causes of MTX interruption were side effects (66/88=75.00%) (**Table 2**), inefficiency (12/88=13.63%), and other reasons (10/88=11.36%). 85 patients (96.6%) were under corticosteroids (mean dose: 7.59±1.35 mg/day) after stopping MTX.

**Table 2.** Methotrexate discontinuation by side effect among rheumatoid arthritis patients.

**Side effect**	**Number (n=66)**	**Percentage (%)**
**Gastrointestinal toxicity**	**40**	**60.6**
Nausea	36	54.5
Vomiting	3	4.5
Vomiting and diarrhea	1	1.5
**Hepatotoxicity**	**18**	**27.3**
Cytolysis	17	25.8
Cholestasis	1	1.5
**Hematologic toxicity**	**4**	**6.1**
Neutropenia	2	3
Leukoneutropenia	2	3
**Pulmonary toxicity**	**2**	**3**
** **Interstitial lung disease	1	1.5
Persistent dry cough	1	1.5
**Skin toxicity**	**3**	**4.5**
Rheumatoid nodules	2	3
Skin allergy	**1**	**1.5**

### Predictive Factors of Methotrexate Interruption using Cox Model Regression Analysis

In univariate analysis, the presence of rheumatoid factor was associated with interruption of MTX while the access to primary education system was identified as a protective factor. There were no differences in BMI, smoking habit, social security, duration of RA, DAS28, ESR, and corticosteroids or NSAIDs intake. In multivariate analysis, the presence of rheumatoid factor (HR 2.24; 95% CI 1.14–5.15; p=0.02) and the access to primary education system (HR 0.37; 95% CI 0.16–0.88; p=0.02) were still associated with cessation of MTX (**Table 3**).

**Table 3.** Predictive factors for methotrexate interruption using Cox model regression.

**Characteristics**	**MTX interruption**					
	**Univariate analysis**			**Multivariate analysis**		

	**HR**	**95% CI**	***p*** **value**	**HR**	**95% CI**	***p*** **value**
**Rheumatoid factor**	2.08	[0.99–4.39]	0.05^*^	2.24	[1.14–5.15]	0.02
**Educational level (primary)**	0.42	[0.18–0.96]	0.04^*^	0.37	[0.16–0.88]	0.02
**Age > 50 years**	0.93	[0.60–1.44]	0.75			
**Sex**	1.53	[0.79–2.99]	0.20			
**ACPA > 10× ULN**	1.20	[0.67–2.13]	0.52			
**Social security**	0.83	[0.46–1.50]	0.55			
**Duration of RA**	0.99	[0.99–1.00]	0.06			
**BMI > 25**	1.02	[0.79–1.30]	0.86			
**Smoking**	1.80	[0.18–9.40]	0.70			
**ESR > 40**	0.85	[0.68–1.07]	0.18			
**DAS28-ESR > 3.2**	1.01	[0.79–1.27]	0.95			
**Corticosteroid treatment**	1.04	[0.58–1.86]	0.87			
**NSAIDs intake**	1.07	[0.80–1.26]	0.92			

ACPA, anti-citrullinated protein antibodies; BMI, body mass index; CI, confidence interval; DAS, disease activity score; ESR, erythrocyte sedimentation rate; HR, hazard ratio; MTX, methotrexate; NSAIDs, non-steroidal anti-inflammatory drugs; RA, rheumatoid arthritis; ULN, upper limit of normal.

* *p* value ≤ 0.05.

## DISCUSSION

In this study, we observed a relatively high therapeutic maintenance level of MTX (over 50%). We also noted that the primary reason for stopping MTX treatment in our population was due to side effects, especially gastrointestinal toxicity, which is contrary to other csDMARDs for which the primary cause of interruption was inefficiency.^13^

The evaluation of the tolerance of MTX and the analysis of its therapeutic maintenance level in RA has not been well studied. Our report was the first multicentre study that analysed the therapeutic maintenance level of MTX and the predictive factors for stopping this drug in Morocco. To our knowledge, this was the third national study conducted to evaluate the therapeutic maintenance level of MTX in RA patients and the second national study to identify predictive factors for MTX discontinuation in RA. Previous international and Maghreb studies have shown that the therapeutic maintenance level of MTX is higher at 1 year, 2 years, and 5 years, which is comparable with our results.^14–19^ A previous study conducted in our hospital that included 100 RA patients over a 20-year period reported a therapeutic maintenance level of MTX of 76% at 1 year, 63% at 2 years, and 45% at 5 years,^20^ and a second study conducted by the rheumatology team of the Med V University Hospital in Marrakech^21^ with 85 RA patients over a period of 8 years also reported similar MTX maintenance levels of 73% at 1 year, 59% at 2 years, and 39% at 5 years. In a French study, Sany et al. found therapeutic maintenance levels of MTX at 73% at 1 year, 65% at 2 years, and 46% at 5 years.^17^

Although there has been a decrease in the therapeutic MTX maintenance level over time, it has still been proven to be superior to csDMARDs. Indeed, the maintenance level of other DMARDs remains low, not exceeding 20% at 2 years and 8% at 5 years.^12^

In a long-term observational study including 428 patients with early RA, the aim was to investigate the survival of Cs DMARDS. The highest therapeutic maintenance level was observed in patients treated with MTX, followed by cyclosporine A, with no significant difference between them.^22^ A study of maintenance therapy with MTX compared with leflunomide for patients with RA, the probability of maintaining MTX monotherapy was higher (81%) than maintaining LEF monotherapy (55%) (p = 0.025).^23^

The occurrence of side effects during treatment of RA with MTX was the major limiting factor in therapeutic maintenance.^24–29^ Similar to other studies,^30,31^ the main reason for discontinuation of MTX in our population was the occurrence of side effects in 75% of patients, mostly represented by gastrointestinal toxicity and hepatotoxicity in 66.6% and 27.3% of cases, respectively. In rare instances haematological toxicity and respiratory impairment was also observed. Compared with data from the literature, where the frequency of occurrence of side effects ranges from 29.3–95.3%,^29,32–34^ our results remained high. Taking account into the advanced age of our population which may decrease the tolerance to MTX and the absence of folic acid in Morocco, the frequency of occurrence of side effects in our study group may be justified.

In contrast to other DMARDs, where inefficiency plays a major role in drug interruption,^13^ the percentage of MTX discontinuation for inefficiency in the literature ranges from 2–17.2%.^18, 34–37^ This is consistent with our results where MTX was stopped for inefficiency in only 13.63% of patients.

Discontinuation of MTX treatment was independent of efficiency or side effects in 10.36% of patients who stopped MTX, which is in line with results from two other Moroccan studies.^20,21^ These results may be explained by the low socio-economic level and the limited number of patients adhering to a social security system on the one hand, and illiteracy and the low educational level of the patients on the other. These findings reinforce recommendations of scientific societies for the management of RA, which stress as a first principle the importance of information and education of the patient.^2,3^ The few studies that have attempted to identify the predictive factors that are associated with discontinuation of MTX have been contradictory. In our study, several factors appear to influence the continuation of MTX treatment. Among the predictive factors for MTX interruption was presence of rheumatoid factor, while the access to primary education system was identified as a protective factor. For Bencharif et al.^19^ female sex and the erosive nature of RA emerged as predictors of MTX cessation. Advanced age is a classic factor for poor tolerance of MTX in RA.^26,30^ Alarcon et al.^27^ has suggested that the severity of RA may influence the rate of MTX maintenance. Other studies have demonstrated a relation between stopping MTX and the initial level of serum creatinine and white blood cells. Fürst et al.^20^ found that the mean serum creatinine value at the start of treatment with MTX was significantly higher in patients who develop at least one side effect, and an initial value of white blood cells <5,000 elements/mm^3^ was a predictive factor for stopping MTX.

Previous studies suggest that despite the early treatment on RA patient, including by MTX, cigarette smoking is associated with increased disease activity and radiological damage. This will lead to discontinuation of MTX for inefficacy.^38^

A study evaluating SLC19A1 genetic variations on the therapeutic response to MTX in RA patient, based in the role of reduced folate carrier protein (SLC19A1-gene) in the uptake and intracellular accumulation of folates. This study revealed the need of a complex genotypic analysis in order to predict patient response to antifolate treatment.^39^

Our study had some limitations. The national registry database contained some missing data points, particularly with the dose of MTX, routes of administration, biological constants at the time of initiation, and cessation of MTX treatment. Conversely, it had some strong points: it was the first large multicentric study exploring rheumatic disease patients under biotherapy in Morocco and in the north of Africa.

## CONCLUSION

We have demonstrated that the therapeutic maintenance of MTX in our sample Moroccan population was satisfactory and comparable to other series. The causes of MTX cessation were diverse but represented mainly by the occurrence of a side effect. Aside from inefficiency and adverse events, cessation of MTX was mainly due to lack of information and education. This data may be helpful to medical practitioners who are considering prescribing MTX, especially in light of its low toxic profile. Moreover, educating patients about the advantages and disadvantages of MTX treatment and sharing of the therapeutic decision between the rheumatologist and patient are important and necessary for the adequate management of RA.

## Data Availability

The datasets are available from the RBSMR registry of the Moroccan Society of Rheumatology.

## ABBREVIATIONS

ACPA: Anti-citrullinated Peptide Antibody
BMI: Body mass index
CI: Confidence interval
CRP: Protein-C- Reactive
CsDMARDS: Conventional Disease Modifying anti-Rheumatic Drugs
DAS28: Disease Activity Score
ESR: Sedimentaion rate
HAQ-DI: Health assessment questionnaire disability index
HR: Hazard ratio
IQR: Interquartile range
MTX: Methotrexate
RA: Rheumatoid arthritis
RBSMR: Registre de biothérapie de la société marocaine de rhumatologie :Biotherapy register of the Moroccan society of rheumatology
SD: Standard deviation
SpA: Spondyloarthritis
